# Hg^2+^ Optical Fiber Sensor Based on LSPR Generated by Gold Nanoparticles Embedded in LBL Nano-Assembled Coatings

**DOI:** 10.3390/s19224906

**Published:** 2019-11-10

**Authors:** María Elena Martínez-Hernández, Javier Goicoechea, Francisco J. Arregui

**Affiliations:** 1 Department of Electrical, Electronic and Communication Engineering, Universidad Publica de Navarra, Edif. Los Tejos, Campus Arrosadía, 31006 Pamplona, Spain; javier.goico@unavarra.es (J.G.); parregui@unavarra.es (F.J.A.); 2 Institute of Smart Cities (ISC), Universidad Publica de Navarra, Campus Arrosadia, 31006 Pamplona, Spain

**Keywords:** fiber optic sensor, gold nanoparticles, localized surface plasmon resonance, mercury, ppb

## Abstract

Mercury is an important contaminant since it is accumulated in the body of living beings, and very small concentrations are very dangerous in the long term. This paper reports the fabrication of a highly sensitive fiber optic sensor using the layer-by-layer nano-assembly technique with gold nanoparticles (AuNPs). The gold nanoparticles were obtained via a water-based synthesis route that use poly acrylic acid (PAA) as stabilizing agent, in the presence of a borane dimethylamine complex (DMAB) as reducing agent, giving PAA-capped AuNPs. The sensing mechanism is based on the alteration of the Localized Surface Plasmon Resonances (LSPR) generated by AuNPs thanks to the strong chemical affinity of metallic mercury towards gold, which lead to amalgam alloys.

## 1. Introduction

The presence of mercury in the environment is a real concern nowadays. It is well known that mercury not only causes damage to the environment, but also to human health [1]. It is a highly toxic element known to cause DNA damage, lipid peroxidation, and protein oxidation and deactivation and is also associated with cardiovascular diseases [2]. This has become a priority matter in the European Union (EU) and the United States Environmental Protection Agency (US-EPA) [3], which seek to take actions against diverse harmful agents that attack the environment like solvents, hydrocarbons, pesticides, and heavy ions (mercury among them). Their objective is to improve the determination of human exposure through integrated monitoring of the environment and food [4,5]. In 2006, the International Conference on Chemicals Management adopted the “Dubai Declaration on International Chemicals Management”, the “Overarching Policy Strategy”, and endorsed the “Global Plan of Action”, in which priority attention is given to mercury [6].

Those international institutions have regulated that any water source and aquifer as well as some specific food products should be monitored and controlled in order to guarantee that they have admissible levels of a series of dangerous contaminants [7], but this task cannot be done nowadays because these tests would require unaffordable costs and complexity. That is why there are many research works that are focused on finding simpler, better, and more accurate ways to detect mercury, where the biggest challenge is to obtain quick, cost-effective and accessible results. 

To solve this problem, new methods and perspectives with the use of different sensor devices have been reported. Classical approaches include the monitoring of electrochemical reactions, using techniques like galvano-static techniques, impedance measurement, electrochemiluminescence, and others [1,2,3]. However, most suffer some reproducibility and stability problems [4]. 

Among the different sensing materials, gold nanoparticles are one of the most interesting materials for optical sensing applications [5], because of their stability, compatibility with the aqueous medium, easy surface functionalization along with miniaturization [6], and their optical properties. When gold nanoparticles interact with light, there is a resonant light-matter energy coupling known as Localized Surface Plasmon Resonances (LSPR) which can be used as a sensing signal [7,8,9,10]. The LSPR occurs thanks to the energy transfer from incident light to certain collective oscillation modes of the free electrons within the nanoparticles that creates an intense optical absorption band. The location of this resonant peak in the visible or infrared region depends on multiple factors such as shape, size, aggregation state, distribution or interaction of the nanoparticles [9]. The consequences of exciting the LSPR are the selective absorption of certain excitation wavelengths and the generation of locally enhanced or amplified electromagnetic fields (EM) on the surface of the nanoparticles and their resonant condition is very sensitive to refractive index variations of the close surrounding medium and the surface chemistry of the nanoparticles [11]. Some studies base their sensing mechanism on the variation of the optical absorbance intensity of the LSPR bands [12,13,14] of the gold nanoparticles simply due to their surface interaction with mercury ions. Sensors of this type can have low detection limits [15,16]. Furthermore some of the reported works require the use of additional measuring techniques such as ellipsometry [17], SPR reflectometry [18] or involving biological reactions, such as aptamer-based recognition [15], allowing highly sensitivities (LODs around), but increasing the sensors complexity and their cost. Those approaches suppose a limitation for the practical use of such sensors, and the development of more robust, simple and effective sensitive coatings is still a challenge nowadays. 

Fiber optic sensors can be a simpler and powerful alternative to these nanoparticles dispersions analysis because have small size, electromagnetic immunity, electrically passivity, and biocompatibility [19]. One of the most common approaches to create optical fiber sensors is the immobilization of the sensitive material onto the surface of the optical fiber. In this manner, the guided light is altered by the interaction with the sensitive material whose optical properties can be affected by the presence of the target to be measured. Therefore, the photonic signal traveling through the fiber will be also modified, which constitutes one of the most common transduction principles of optical fiber sensors for chemical measurements [20]. So far, gold nanoparticles are the most popular solution for the development of highly-sensitive mercury fiber optic sensors, thanks to their stability, small size, low cost, and outstanding optical properties. In most of the approaches, additional molecules or biomolecules are needed to cause this LSPR variation, such as the tendency of mercury to form complexes with certain proteins [13] or the use IgG–anti IgG as bioreceptor–analyte pair [14]. Other sensors study the change of LSPR resonance wavelength. For example, it has been reported the plasmon-coupling effect in gold nanoparticles core-satellites nanostructures linked by thymine(T)-rich DNA hybridization [21]. It is known that the shape and distribution of gold nanoparticles can generate changes in the LSPR, causing wavelength shifts [11]. The process of Au–Hg alloy is able to modify the shape of gold nanoparticles causing changes remarkable blue shifts. Such changes occurred because of the chemical modification of the nanoparticles near their surface (Hg–Au amalgam formation) modify their effective size and shape [22] altering the LSPR resonant condition.

In this work, it is proposed the embedding of gold nanoparticles in a polymeric matrix that allow the interaction with mercury ions (Hg^2+^). The sensing mechanism is based on the strong chemical affinity of metallic mercury (Hg^0^) towards gold [18] to form stable amalgam-like alloys [17], and consequently altering the LSPR resonance of the gold nanoparticles, therefore, providing a wavelength-based sensing signal. It has been already reported that the reaction of metallic mercury on the surface of the gold nanoparticles can cause the change of their shape, affecting to the LSPR resonance conditions [22].

The layer-by-layer nano-assembly technique is used here for such embedding of the metallic nanoparticles in the matrix that can facilitate the gold-mercury interaction. This sensing mechanism is simpler than the previous approaches reported in the literature and does not involve the utilization of auxiliary biomolecules with the gold nanoparticles. 

## 2. Materials and Methods

### 2.1. Materials

The polymer poly (allylamine hydrochloride) (PAH) (Mw~15.000) was used as polycation during the LbL process. For the synthesis of AuNPs it was used poly (acrylic acid) (PAA) 35 wt% solution in water, Borane dimethylamine complex (DMAB) and Gold (III) chloride trihydrate. The pH of the solutions were adjusted using HCl and NaOH. The mercury samples were prepared with Mercury (II) chloride (HgCl_2_) in buffer phosphate. For the buffer solutions it was used sodium phosphate dibasic (Na_2_HPO_4_) and sodium phosphate monobasic (NaH_2_PO_4_). Piranha solution was also used, which is the combination of sulfuric acid (H_2_SO_4_) with hydrogen peroxide (H_2_O_2_), 3:1 ratio. All materials were supplied by Sigma Aldrich and aqueous solutions were prepared using ultrapure water with a resistivity of 18.2 MΩ·cm.

### 2.2. Synthesis Method of the PAA-Capped AuNPs

There are other works that describe different synthesis routes for metallic nanoparticles of various morphologies [23,24,25,26]. In this case, to AuNPs synthesis it was used a chemical reduction route carried out in water-based solutions in which the PAA act as a stabilizer [23]. Gold nanoparticles have been prepared by adding 20 mL of HAuCl_4_·3H_2_O (5 mM) to 120 mL of PAA (10 mM). This solution was stirred for 2 h. Afterwards 1 mL of fresh DMAB (0.1 M) solution was added under vigorous stirring, and the reaction was left overnight. All operations were performed at room conditions. UV-VIS absorption spectra of the synthesized nanoparticles dispersions were characterized using a Jasco V-630 spectrophotometer. The UV-VIS absorption spectra of the PAA-AuNP dispersions showed a LSPR absorption band centered at 540 nm. Transmission electron microscopy (TEM) has been used to determine the morphology of the AuNPs, resulting in spherical shape particles, with a diameter ranging from 10 to 20 nm [23].

### 2.3. Optical Detection Setup

Optical fiber sensors were made from multimode optical fibers 200 μm-core diameter with polymeric cladding, 0.39 NA (THORLABS FT 200EMT). The sensor structure was based on the mechanical removal of the acrylate cladding of a segment of approximately 2 cm of the optical fiber. This removal was performed with the help of a few drops of dry acetone and a blade, exposing the bare optical fiber core, in its entire cylindrical section. Subsequently, this optical fiber segment was immersed for 5 min in piranha solution to eliminate the acetone that could remain. The ends of the optical fiber were terminated using temporary SMA connectors (THORLABS BFT1). The sensor was excited from one of the connectors with a halogen white source and the other end collect the optical response with a CCD spectrometer (HR4000-UV Ocean Optics). 

### 2.4. Layer-By-Layer Nano-Assembly

**Figure 1:**
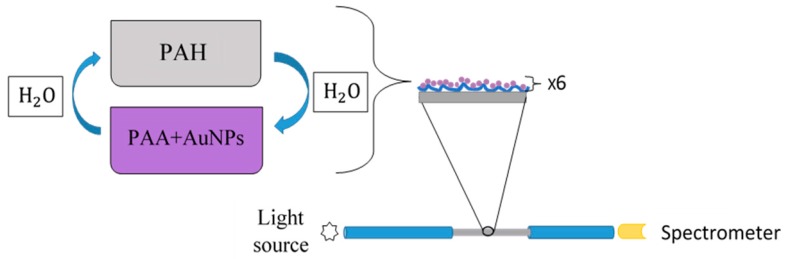
Layer-by-layer nano-assembly built-up of the sensitive coatings. Construction of fiber optic sensor with (PAH/PAA+AuNPs)_6_ over a cladding-removed 200 μm-core optical fiber.

Using layer-by-layer nano-assembly (LbL) it is possible the deposition of oppositely charged polyelectrolyte ultra-thin layers by dipping the substrates into a sequence of solutions. A solution of PAH (10 mM) was used as polycationic solution, and PAA-capped AuNPs (PAA-AuNPs) dispersion was used as polyanion. The optical fiber substrates were immersed into each charged solution for 5 min. After every polyelectrolyte adsorption step it is necessary to rinse the assembly in ultrapure water with same pH of the polyelectrolytes [24,25]. Each polycation/polyanion layer combination is called bilayer. In this work, a total of six bilayers of (PAH/PAA+AuNPs) are deposited onto the cladding removed optical fiber segment (Figure 1). All solutions were adjusted to pH 7. Before starting the deposition of layer by layer, the entire fiber segment where the cladding was removed was immersed in KOH (1M) for half an hour to achieve substrate surface electrostatic charge 

### 2.5. Mercury Samples

Phosphate buffer (PB) was prepared dissolving 2.198 g of Na_2_HPO_4_ in 400 mL of ultrapure water. This solution was stirred for 15 min, then 0.62 g of NaH_2_PO_4_ was added and 100 mL of ultrapure water, and stirred for 15 min, obtaining a pH = 7.6. The different concentrations of Mercury (II) chloride (HgCl_2_) were dissolved in the PB. Different concentration mercury samples must be in metallic form to interact with the AuNPs, consequently, before exposing the optical fiber sensor to the mercury ions, it is necessary to reduce Hg^2+^ to Hg^0^ using DMAB (12 mL of a freshly prepared DMAB stock solution (0.1 M) as reducing agent. The reaction was stirred at room temperature conditions for 2 h (kept away from direct sunlight). The Hg concentrations analyzed were 1, 2, 4, 8, 10, and 20 ppb. All samples were kept under stirring until the moment of measurement.

In order to vary only the mercury concentration and keep the rest of parameters constant, the DMAB amount is corrected for every sample just adding a certain amount of blank PB stock solution. For every measurement, the optical fiber sensor was immersed into the Buffer PB + DMAB solution prior to the exposure to the mercury ion stock solutions, in order to get a stable baseline for the latter mercury detection.

### 2.6. Sensors Regeneration

It is known that nitric acid forms highly instable complexes with Hg^2+^ and favors the separation of mercury from gold nanoparticles [26]. The regenerating solution was prepared starting from a stock PB (pH 7.6) and HNO_3_ was added dropwise until the pH was lowered to 4.6 and the dissolution was keep at a constant temperature of 55 °C.

### 2.7. Data Processing

During the immersion in the mercury solutions all spectra were recorded continuously and the LSPR maxima were estimated using a Matlab^®^ algorithm. This provides live information about the time response of the sensors. The results obtained will be estimated by their dynamic response as a way to obtain parameters for rapid estimation before the responses obtained from the sensor.

### 2.8. Cross-Sensitivity to Other Metals

There are other metals whose presence in the organism is necessary because they are involved in biological functions, however, when they exceed a certain threshold they can be considered toxic, among them we can find zinc and nickel [27]. Consequently it is very important to characterize the mercury sensor cross-sensitivity against other metal ions such as Fe^2+^, Ni^2+^, Pb^2+^, Cd^2+^, and Zn^2+^. All the solutions were prepared under the same conditions as the mercury samples. All ionic species for the cross-sensitivity test has been set to the maximum concentration used with the mercury (20 ppb). This concentration of the other ionic species are significantly higher than the limits required by the regulation [28] like for Fe (6.2 ppb), Cd (3.4 ppb) and Zn (1.8 ppb).

## 3. Results and Discussion

### 3.1. Effects of Hg^0^ on AuNPs in Dispersion

Prior to the construction of the fiber optic sensor, a preliminary study was made in order to characterize the effects of mercury on the optical properties of the gold nanoparticles in dispersion. It is known the Hg^0^ can be bonded onto the surface of Au-based nanomaterials to form a solid amalgam-like alloy [29,30]. 

**Figure 2:**
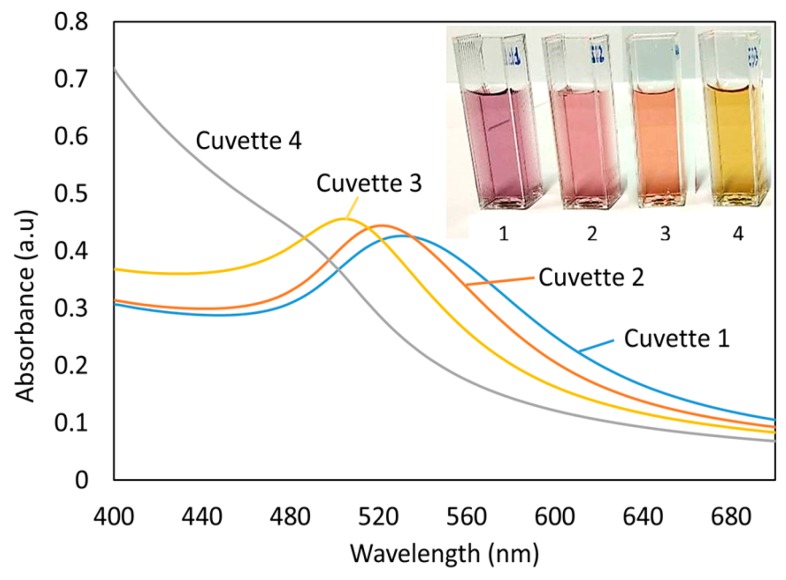
Change of Localized Surface Plasmon Resonances (LSPR) wavelength of PAA-AuNP dispersion with different Hg concentration. Cuvette 1: as prepared only with AuNPs. Cuvette 2: PAA-AuNP + DMAB. Cuvette 3: PAA-AuNP + DMAB and mercury (2.7 ppb). Cuvette 4: PAA-AuNP + DMAB and mercury (27 ppb).

Samples in dispersion were analyzed with different Hg concentration and keeping constant the volume and concentration of AuNPs solutions. US-VIS spectra of the dispersions showed a dramatic change of LSPR resonance wavelength clearly seen with the naked eye as a color change (Figure 2). 

In Figure 2 it is shown the UV-VIS spectrum of cuvette 1 that contain only a PAA-AuNPs dispersion as prepared that shows an violet-reddish color. The spectrum shows the typical LSPR attenuation band centered in 540 nm which is compatible with the synthesis routes available in the literature. Since it is necessary to reduce mercury ions to their metallic form (Hg^0^) to enable the amalgam interaction, cuvette 2 is equal to cuvette 1, except that 200 μL of 0.1 M DMAB were added. Here it is observed a slight blue shift of the LSPR resonance wavelength of 8 nm, that remained stable in time. This LSPR variation is probably due to the modification of the polymeric PAA stabilization cap thanks to the interaction with the DMAB. For lower mercury concentrations such as 2.7 ppb (cuvette 3), the displacement also occurs, but to a lesser extent, in 18 nm respect to the cuvette 2 that is the optical reference with no mercury. When 300 μL of Hg (10^−3^ M) were added to the dispersion keeping the same concentration and volume of PAA-AuNPs and DMAB solution, it was obtained 27 ppb of mercury concentration and the LSPR resonance experimented a stronger blue shift, almost disappearing, yielding a clear yellowish color.

In the synthetic process of AuNPs, the reduction of gold ions ($Au^{3+}$) to gold nanoparticles ($Au^{0}$) is possible thanks to the use of a protective agent (PAA), which contributes to control the shape and size of the resultant nanoparticles, preventing their agglomeration or precipitation in the colloidal solution and the DMAB that acts as a reducing agent [9]. The small displacement of LSPR resonance wavelength that occurred in the case of cuvette 1 as a result of interaction between AuNP-PAA with the additional DMAB present in the sample solutions (with no mercury in cuvette 1). This LSPR wavelength shift could be induced by the refractive index variation in the optical fiber immersion media. However, cuvettes 2, 3, and 4 have the same concentrations of AuNP-PAA and DMAB, they only differ in a very small mercury concentration that induces a more severe LSPR resonance displacement thanks to the chemical modification of the AuNPs.

### 3.2. Obtaining the AuNPs LSPR onto the Fiber Optics

**Figure 3:**
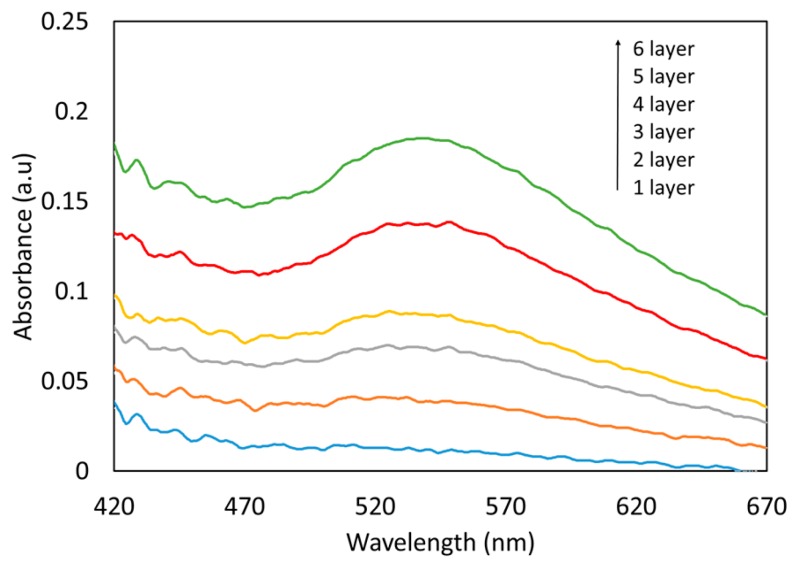
Absorption spectra of LSPR resonance wavelength for every layer of (PAH/PAA+AuNPs) deposited on 200 µm-core optical fiber.

The absorbance of the (PAH/PAA+AuNPs)_n_ coating onto the optical fiber was registered during the LbL process. With every bilayer increment the absorbance spectrum show an increasing of the intensity around the 540 nm wavelength suggesting a homogeneous growth of the LbL coating. After an optimization study of the LbL process it was found that using six bilayers it is possible to obtain a well-defined LSPR absorption band (Figure 3) consequently this number of bilayers was kept constant for all the sensors in this work.

The absorbance of the LbL (PAH/PAA+AuNPs)_n_ films (being n the number of bilayers) is shown in Figure 3. The absorbance spectra confirm the existence of an absorption band centered at 540 nm, which corresponds to the LSPR of the AuNPs. This demonstrates that the absorption band of the coatings matches with that of the AuNPs dispersion initially synthesized by chemical reduction seen in Figure 2 (cuvette 1).

### 3.3. Detection of Mercury Ions with Fiber Optic Sensor

**Figure 4:**
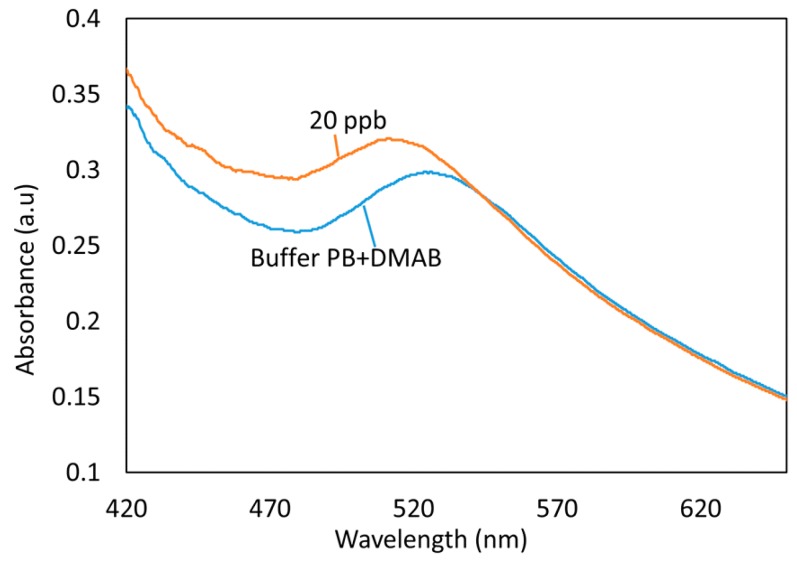
Displacement in wavelength of the LSPR for 20 ppb of mercury concentration.

In Figure 4, it is shown an initial immersion of the sensor in the Buffer PB + DMAB solution, it was registered a small displacement of LSPR resonance wavelength (approximately 5 nm) and after a few minutes it remained stable. In this work, all sensors were kept in this solution for one hour in order to have a stable baseline for the later mercury detection stage. Nevertheless, shorter immersion times could be also acceptable. After the sensor it was immersed in a 20 ppb mercury sample and there was a variation of absorption with respect to the condition of the baseline. In addition, for the mercury concentration of 20 ppb there is a change in the LSPR resonance wavelength of 15 nm with respect to the Buffer PB + DMAB solution.

**Figure 5:**
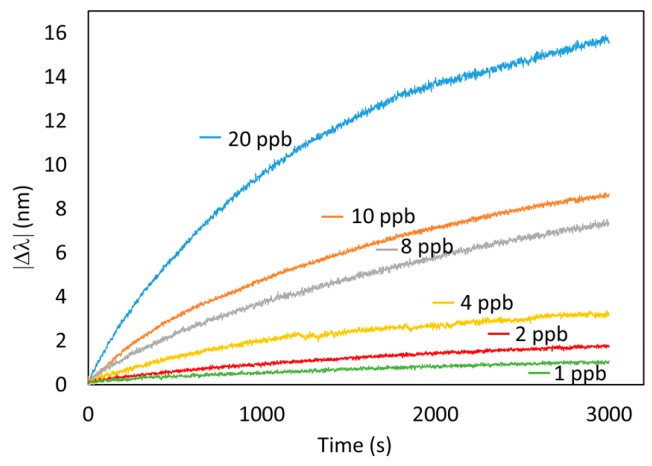
Dynamic response of the sensors to different Hg concentration, ranging from 20 ppb to 1 ppb.

Different sensors were fabricated with the same materials and methods mentioned in Section 2, and each one was used to detect a particular mercury concentration (shown in Figure 5). Although the wavelength shift of the LSPR band was easily visible in a few seconds for the highest mercury concentration, the (PAH/PAA+AuNPs)_6_ sensors showed a settling time of nearly 3000 s (from 10% to 90%). Consequently, all the sensors were immersed in the mercury solution for 50 min. All sensors’ LSPR bands experimented a blue-shift when exposed to mercury. The absolute wavelength shift increases with the mercury concentration; for 20 ppb of mercury solution, the LSPR maximum wavelength change is 16 nm, and for 1 ppb is 1.11 nm. 

**Figure 6:**
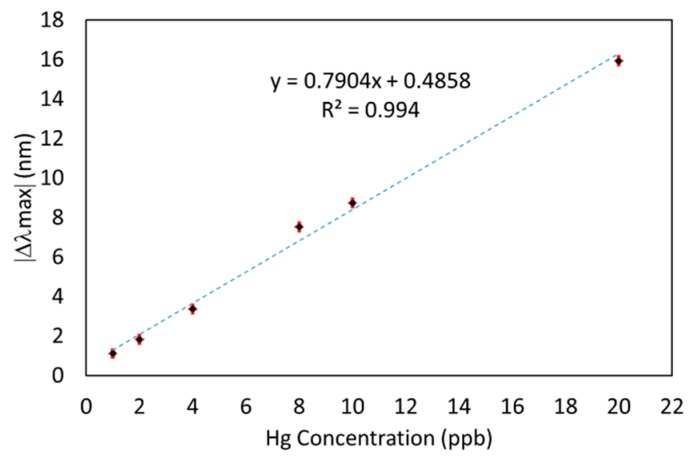
Maximum variation in wavelength (Δλmax) for each mercury concentration (1, 2, 4, 8, 10, and 20 ppb) after 3000 s.

This wavelength-based response can be seen in Figure 6, where it is shown the maximum variation in wavelength ǀΔλmaxǀ for each mercury concentration and the linear fitting.

From the continuous data acquisition of the baseline during the immersion in Buffer PB + DMAB (0 ppb), it is possible to calculate the standard deviation (σ). The limit of detection (LOD) of the sensor can be estimated as 3σ, that is 0.147 nm, which is equivalent to 0.7 ppb, which is below the 2 ppb detection limit stated by the US-EPA and 1 ppb for the EU [29,30].

**Figure 7:**
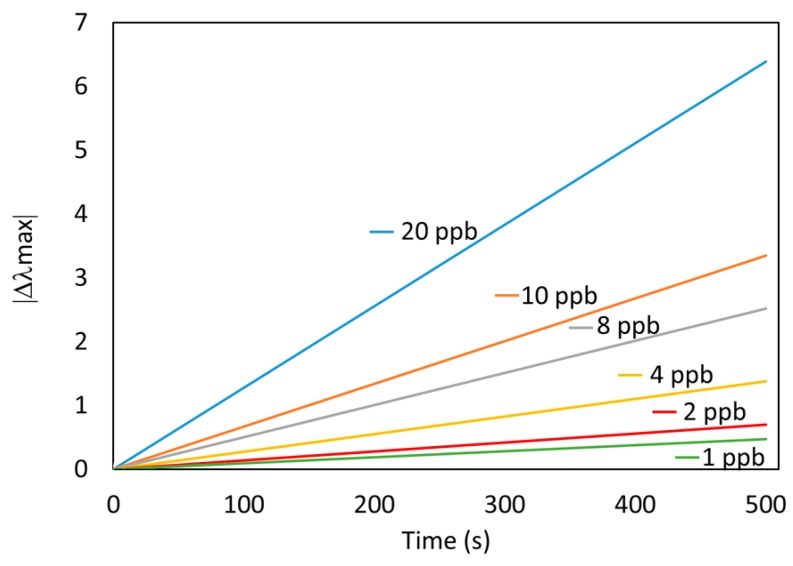
Fast estimation of the optical fiber sensor response using the slope of the linear fitting of the samples of the first 500 s. The Hg concentration has been varied from 1 to 20 ppb.

The results presented so far are accurate enough to provide reliable measurements of aqueous samples without any further chemical or biological agent, and they could be performed in the field. Nevertheless, the sensors still need relatively long time measurements. In order to overcome this, a measurement technique is proposed to obtain faster measurements. In this sense, the values of the slopes of each dynamic curve (Figure 5) could be used as a fast estimation parameter. In Figure 7, the slope of the sensor’s response approximated by the linear fitting of the first 500 s (roughly 8 min) is plotted for every Hg concentration.

**Figure 8:**
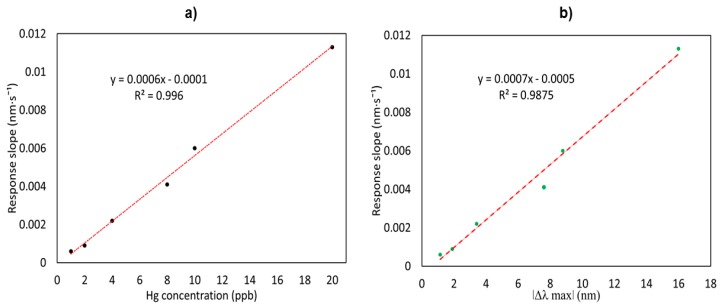
(**a**) Slopes of the linear fitting of the first 500 s vs. mercury concentration. (**b**) Correlation between the mercury measurement using the absolute wavelength shift of the LSPR, and the fast slope estimation.

As can be observed, the value of the slope increases with the increase in mercury concentration, getting lineal response (Figure 8a). In Figure 8b it is shown the high correlation between the absolute wavelength shift and the slope of each curve, meaning that it can be reliably used as a fast response estimator. These results allow estimating the behavior of the sensor in different mercury concentrations in a faster way, after 500 s.

### 3.4. Sensor Regeneration

**Figure 9:**
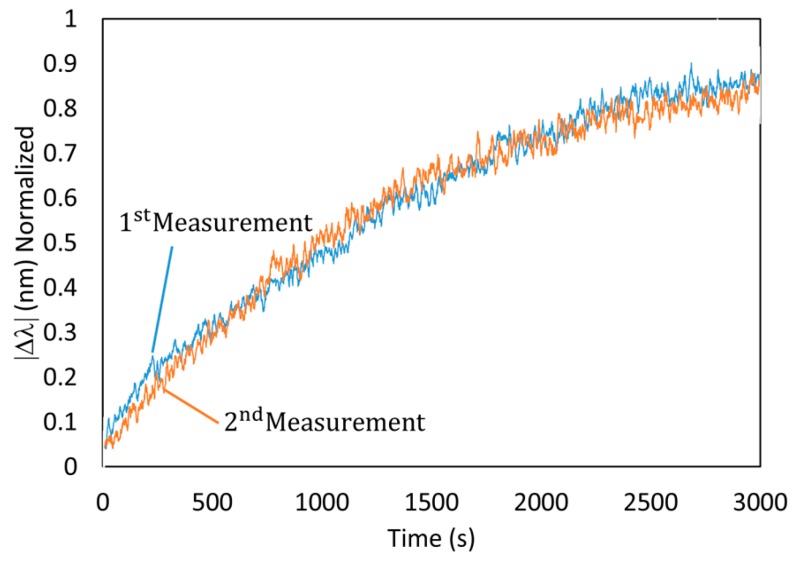
Regeneration of the optical fiber sensor. Repeatability of the sensor under 20 ppb of mercury after its regeneration in a dilute nitric acid solution. The final wavelength-shift is very similar in both cases, and the response slope is even more stable.

Another critical aspect is the reusability of a single optical fiber sensor for multiple measurements. In fact, it is possible to regenerate the sensor in a HNO_3_ solution, the sensor was deposited in the solution mentioned in Section 2.6 for 1 h. During the immersion in the regenerating solution it was observed a red shift of the LSPR resonance wavelength, which is a similar to the first reaction curve. After regeneration the sensor was submerged again in a second Hg dissolution of 20 ppb. As can be seen in Figure 9, the comparison of two measurements of the same optical fiber sensor against two different samples of mercury (20 ppb) is represented. The first measurement corresponds to the freshly fabricated sensor that was deposited in a first mercury sample (20 ppb). After reacting to mercury was deposited in the regenerative solution that allowed the sensor to recover the initial conditions, so it was deposited in a new sample of mercury (20 ppb), thus obtaining the second measurement, yielding a very similar wavelength shift as in the first measurement.

### 3.5. Cross Sensitivity

**Figure 10:**
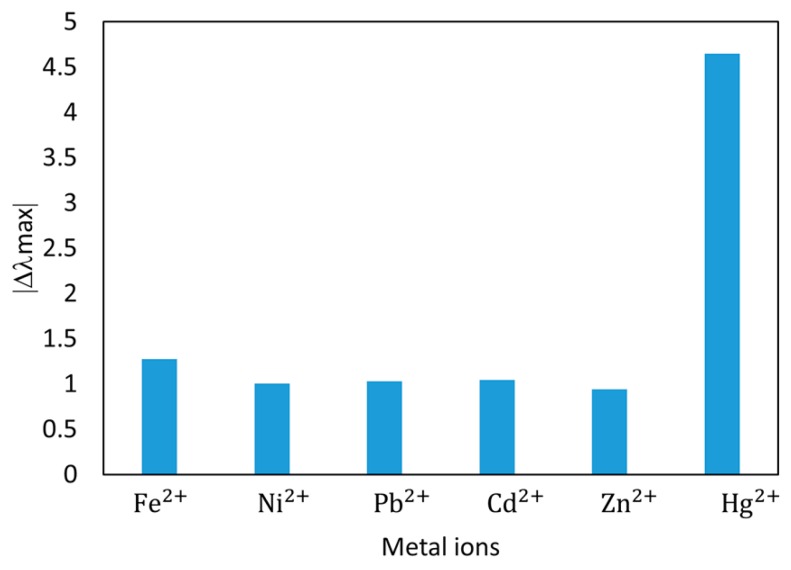
Selectivity analysis against the most common heavy ions. The measurements were carried out using the same sampling preparation process, and with the same concentration (20 ppb).

Finally, the selectivity of optical fiber sensor against different heavy metal ions (Fe^2+^, Ni^2+^, Pb^2+^, Cd^2+^, and Zn^2+^) is also studied the same sensor has been exposed to the same concentration (20 ppb) of the different metal ions, and all solutions were prepared using the same protocol as in the previous mercury tests (PB + DMAB @pH 7.6). The results of the final wavelength shift after the immersion in the different ion solutions are showed in Figure 10. 

As it can be seen in Figure 10, the proposed optical fiber sensor showed a significantly higher response to the presence of mercury compared to the other metal ions, nearly 400% higher than the second more reactive cross-contaminant (in this case iron) enjoying a high selectivity towards mercury. 

The metal ions analyzed have detection limits allowed in water higher than of mercury [28], for example Ni (15 ppb), Fe (6.2 ppb) among others. Therefore, in normal samples, our sensor would be more selective to mercury than to other metal ions. A further study would require the evaluation of the present devices in real aquifer water samples, but this is out of the scope of the present contribution. This work presents a competitive approach for a mercury optical fiber sensor, with a simple and direct measurement of mercury in water.

## 4. Conclusions

In this work, a simple and highly sensitive mercury optical fiber sensor has been proposed. Its sensing mechanism is based on the wavelength shift of the AuNPs LSPR, thanks to the strong chemical affinity of mercury towards gold. The gold nanoparticles were obtained by synthesis method of the PAA-capped AuNPs, using PAA as a stabilizing agent and DMAB as reducing agent. The LBL nano-assembly technique was used for the incorporation of gold nanoparticles onto optical fiber in a stable sensitive thin-film, (PAH/PAA+AuNPs)_6_. The absolute wavelength-shift was a reliable and robust signal with a relatively long response time of around one hour. In order to obtain faster measurements, the slope of the wavelength variation proved to give reliable results in only 8 min. It is possible to reuse the sensor, something that reduces costs and manufacturing time. In addition, this sensor showed low cross sensitivity towards other metal ions. It was achieved a limit of detection of 0.7 ppb, which is lower than the standard limits recommended by the European Union (1 ppb) and US-EPA (2 ppb). The sensor proposed in this work could be competitive alternative for mercury detection, a problem of global concern.
